# Automated assembly of a reference taxonomy for phylogenetic data synthesis

**DOI:** 10.3897/BDJ.5.e12581

**Published:** 2017-05-22

**Authors:** Jonathan A. Rees, Karen Cranston

**Affiliations:** 1 Duke University, Durham, United States of America

**Keywords:** taxonomy phylogeny automation pipeline synthesis

## Introduction

Any large biodiversity data project requires one or more taxonomies for discovery and data integration purposes, as in "find occurrence records for primates" or "find the taxon record associated with this sequence" (Page 2008). *Examples* of such projects are GBIF (Edwards 2004), which focuses on occurrence records, and NCBI (Federhen 2011), which focuses on genetic sequence records. Each of these projects has a dedicated taxonomy effort that is responsive to the project's particular needs. We present the design and application of the Open Tree Taxonomy, which serves the Open Tree of Life project, an aggregation of phylogenetic trees with tools for operating on them. ([Bibr B3595426][Bibr B3621177], Redelings and Holder 2017, Open Tree of Life project 2017). In order to meet Open Tree's project requirements, the taxonomy is an automated assembly of ten different source taxonomies. The assembly process is repeatable so that we can easily incorporate updates to source taxonomies. Repeatability also allows us to easily test potential improvements to the assembly method. Information about taxa is typically expressed in databases and files in terms of taxon names or 'name-strings' (Patterson 2014). To combine taxonomies it is therefore necessary to be able to determine name equivalence: whether or not an occurrence of a name-string in one data source refers to the same taxon as a given name-string occurrence in another. Solving this equivalence problem requires that we distinguishing occurrences that only coincidentally have the same name-string (homonym sense detection), and unify occurrences only when evidence justifies it. We have developed a set of heuristics that scalably address this equivalence problem.

### The Open Tree of Life project

The Open Tree of Life project consists of a set of tools for:

- synthesizing summary phylogenetic trees ('synthetic trees') from a corpus of phylogenetic tree inputs (input trees)
- matching groupings in synthetic trees with higher taxa (such as Mammalia)
- supplementing synthetic trees with taxa obtained only from taxonomy.

**Figure 1.**
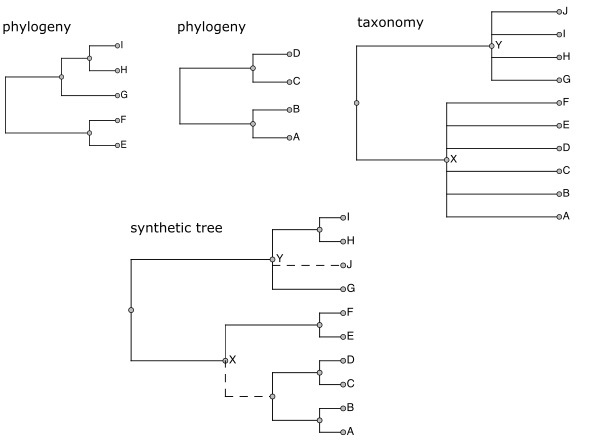
Role of taxonomy in assembly of the Open Tree of Life synthetic phylogenetic tree. Dotted lines in the synthetic tree are those that come only from taxonomy, while solid lines have phylogenetic support. The taxonomy both links disjoint phylogenies and adds taxa not present in input trees.

The outcome is one or more synthetic trees combining phylogenetic and taxonomic knowledge. Fig. 1 illustrates an overview of the process of combining phylogenetic trees and taxonomy, while the details are described in a separate publication (Redelings and Holder 2017).

Although Open Tree is primarily a phylogenetic tree aggregation effort, it requires a reference taxonomy that can support each of these functions.

For synthetic tree synthesis (1), we use the taxonomy for converting OTUs (operational taxonomic units, or 'tips') on input trees to a canonical form. Synthetic tree construction requires that an input tree OTU be matched with an OTU from another input tree **when, and only when, it is reasonable to do so**. This is a nontrivial task because a taxon can have very different OTU labels in different input trees due to synonymies, abbreviations, misspellings, notational differences, and so on. In addition, **a given label can name different taxa in different trees** (homonymy). The approach we take is to map OTUs to the reference taxonomy, so that OTUs in different input trees are compared by comparing the taxa to which they map.

For higher taxon associations (2), we compare the groupings in the synthetic tree to those in the taxonomy.

For supplementation (3), only a relatively small number of described taxa are represented in input trees (currently about 200,000 in the phylogenetic corpus out of two million or more known taxa), so the taxonomy provides those that are not. The large complement of taxonomy-only taxa can be 'grafted' onto a synthetic tree in phylogenetically plausible locations based on how they relate taxonomically to taxa that are known from input trees.

### Reference taxonomy requirements

This overall program dictates what we should be looking for in a reference taxonomy. In addition to the technical requirements derived from the above, we have two additional requirements coming from a desire to situate Open Tree as ongoing infrastructure for the evolutionary biology community, rather than as a one-off study. Following are all five requirements:

1. **OTU coverage**: The reference taxonomy should have a taxon at the level of species or higher for every OTU that has the potential to occur in more than one study, over the intended scope of all cellular organisms.
2. **Phylogenetically informed classification**: Higher taxa should be provided with as much resolution and phylogenetic fidelity as is reasonable. Ranks and nomenclatural structure should not be required (since many well-established groups do not have proper Linnaean names or ranks) and groups at odds with phylogenetic understanding (such as Protozoa) should be avoided.
3. **Taxonomic coverage**: The taxonomy should cover as many as possible of the species that are described in the literature, so that we can supplement synthetic trees as described in step 3 above.
4. **Ongoing update**: New taxa of importance to phylogenetic studies are constantly being added to the literature. The taxonomy needs to be updated with new information on an ongoing basis.
5. **Open data**: The taxonomy must be available to anyone for unrestricted use. Users should not have to ask permission to copy and use the taxonomy, nor should they be bound by terms of use that interfere with further reuse.

An additional goal is that the process should be reproducible and transparent. Given the source taxonomies, we should be able to regenerate the taxonomy, and taxon records should provide information about the taxonomic sources from which it is derived.

No single available taxonomic source meets all requirements. The NCBI taxonomy has good coverage of OTUs, provides a rich source of phyogenetically informed higher taxa, and is open, but its taxonomic coverage is limited to taxa that have sequence data in GenBank (only about 360,000 NCBI species having standard binomial names at the time of this writing). Traditional all-life taxonomies such as Catalogue of Life, IRMNG (Rees 2008), and GBIF meet the taxonomic coverage requirement, but miss many OTUs from our input trees, and their higher-level taxonomies are often not as phylogenetically informed or resolved as the NCBI taxonomy. At the very least, Open Tree needs to combine an NCBI-like sequence-aware taxonomy with a traditional broad taxonomy that is also open.

These requirements cannot be met in an absolute sense; each is a 'best effort' requirement subject to availability of project resources.

Note that the Open Tree Taxonomy is *not* supposed to be a reference for nomenclature; it links to other sources for nomenclatural and other information. Nor is it a place to deposit curated taxonomic information. The taxonomy has not been vetted in detail, as doing so would be beyond the capacity and focus of the Open Tree project. It is known to contain many taxon duplications and technical artifacts. Tolerating these shortcomings is a necessary tradeoff in attempting to meet the above requirements.

### Related work

There are probably about a dozen public all-life taxonomy compilations. The methods of assembly and curation are documented for only a few of these. We assume that most are evolving databases that are extended and maintained by a combination of single-record operations and some amount of *ad hoc* scripting to import material in bulk. The NCBI taxonomy, described in Federhen 2011, is of this type. Catalogue of Life, which documents its method in Species 2000 2017, is different in that it has a divide and conquer approach: it is assembled through systematic grafting of sub-taxonomies received from a network of editors. Because its sub-taxonomies are nonoverlapping, CoL is not a synthesis in the sense used here. The GBIF backbone taxonomy is assembled via automated synthesis of *overlapping* sub-taxonomies, and in that respect is similar to OTT. The GBIF method is as yet unpublished, although some information is available (Döring 2016b, Döring 2016a). Note that OTT builds on GBIF, which in turn builds on CoL.

Had an available all-life taxonomy met all of Open Tree's requirements, the project would have used it. Unfortunately, for each one, there is at least one Open Tree requirement that goes beyond what the taxonomy provides.

An important part of any synthesis method is name-string parsing and matching, and this is the focus of the Global Names Architecture (Pyle 2016). In the phase of work reported here, name parsing and matching are not a priority issue, since exact matches together with synonym records from the source taxonomies provide a provisional solution that has been adequate so far. Going forward, matching will need more attention, and components of the GNA will probably play a role.

The Taxonomy Tree Tool (TTT, Lin et al. 2016) employs a tree merge method similar to the one used here. It seems to be aimed at assisting manual analysis of tree differences. It is not clear from available documentation how one would use it to resolve conflicts in an automated workflow.

## Method

The conventional approach to meeting the requirements stated in the introduction would be to create a database, copy the first taxonomy into it, then somehow merge the second taxonomy into that, repeating for further sources if necessary. However, it is not clear how to meet the ongoing update requirement under this approach. As the source taxonomies change, we would like for the combined taxonomy to contain only information derived from the latest versions of the sources, without residual information from previous versions. Many changes to the sources are corrections, and we do not want to retain information that has been corrected or superseded by a later version of a source.

Rather than maintain a database of taxonomic information, we instead developed a process for assembling a taxonomy from two or more taxonomic sources. With a repeatable process, we can generate a new combined taxonomy version from new source taxonomy versions *de novo*, and do so frequently. There are additional benefits as well, such as the ability to add new sources relatively easily, and to use the tool for other purposes.

In the following, any definite claims or measurements refer to the Open Tree reference taxonomy version 3.0.

### Terminology

- source taxonomy: imported taxonomic source (NCBI taxonomy, etc.)
- workspace: data structure for creation of the reference taxonomy
- name-string: name of one or more taxa, without author information, considered as a sequence of characters, without association with any particular description, or nomenclatural code
- node: a data record intended to correspond to a taxon. Records name-strings, authorship, parent node, optional rank, optional annotations. If a workspace node, it originates from a single source taxonomy, and records its source (provenance) and its alignments to others
- parent (node): the nearest enclosing node within a given node's taxonomy
- tip: a node that is not the parent of any node
- primary name-string: one particular name-string of a node. Each node has exactly one primary name-string
- homonym name-string: a name-string that belongs to multiple nodes within the same taxonomy. This is analogous to the nontechnical meaning of 'homonym' and is not to be confused with 'homonym' in the nomenclatural sense, which only applies within a single nomenclatural code. Nomenclatural homonyms and hemihomonyms (Shipunov 2011) both correspond to homonym name-strings, as do clerical errors where multiple nodes are created for the same taxon
- synonym name-string (of a node): a non-primary name-string
- image (of a node n'): the workspace node corresponding to n'
- *incertae sedis*: node A is *incertae sedis* in node B if A is a child of B but is not known to be disjoint (as a taxon) from B's non-*incertae-sedis* children. That is, if we had more information, it might turn out that A is a member of one of the other children of B.

### Method overview

This section is an overview of the taxonomy assembly method. Several generalities stated here are simplifications; the actual method (described later) is significantly more involved.

We start with a sequence of source taxonomies S1, S2, ..., Sn, ordered by priority. Priority is the means by which conflicts between sources are resolved, and therefore has a profound effect on the outcome of assembly. If a curator judges S to be more accurate or otherwise "better" than S', then S will occur earlier in the priority sequence than S' and its information supersedes that from later sources. Curators (either project personnel or participants in Open Tree workshops and online forums) determine priority based on their taxonomic expertise. Source taxonomies are sometimes split into pieces in order to establish different priorities for different parts. Priority choice by curator is a fragile and subjective aspect of the method, but we could not identify any other information available at scale that could be brought to bear on conflict resolution.

We define an operator for combining taxonomies pairwise, written schematically as U = S + S', and apply it from left to right:

U0 = empty, U1 = U0 + S1, U2 = U1 + S2, U3 = U2 + S3...

The combination S + S' is formed in two steps:

1. A mapping or *alignment* step that identifies all nodes in S' that can be equated with nodes in S. There will often be nodes in S' that cannot be aligned to S.
2. A *merge* step that creates the combination U = S + S', by adding to S the unaligned taxa from S'. The attachment position of unaligned nodes from step 1 is determined from nearby aligned nodes, either as a *graft* or an *insertion*.

**Figure 2.**
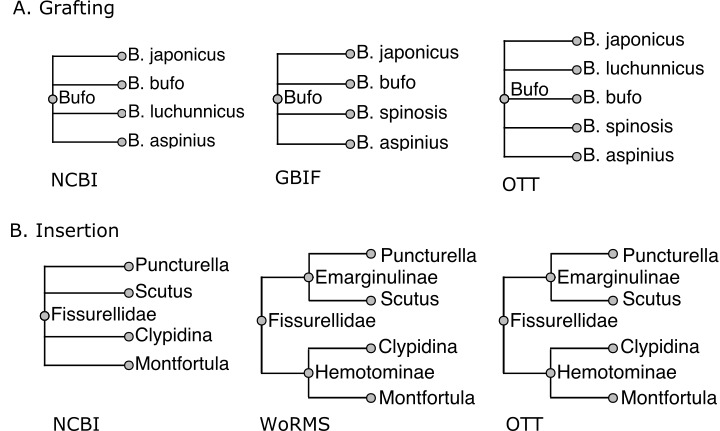
*Examples* of grafting and insertion when combining taxonomies. In both cases, the NCBI taxonomy has higher priority than GBIF. In A (grafting), we assemble the genus *Bufo* across NCBI and GBIF. There is no *B. spinosis* in GBIF and no *B. luchunnicus* in NCBI. Therefore, the *Bufo* in the combined taxonomy has as its children copies of species records from both sources. In B (insertion), WoRMS provides greater resolution of *Fissurellidae* than NCBI taxonomy: it divides the family into subfamilies *Hemotominae* and *Emarginulinae*, nodes that do not exist in NCBI. The subfamilies are 'inserted' in a way that adds information without disrupting existing relationships from NCBI.

*Examples* of these two cases are given in Figure 2.

As a simple example, consider a genus represented in both taxonomies, but containing different species in the two:

S = (b,c,d)a, S' = (c,d,e)a

S and S' each have four nodes. Suppose c, d, and a in S' are aligned to c, d, and a in S. The only unaligned node is e, which is a sibling of c and d and therefore grafted as a child of a. After the merge step, we have:

S + S' = (b,c,d,e)a

One might call this merge heuristic 'my sibling's sibling is my sibling' or 'transitivity of siblinghood'.

This is a very common pattern. Fig. 2 illustrates a real life-example when combining the genus *Bufo* across NCBI and GBIF. There are about 900,000 similar simple grafting events in the assembly of OTT.

The other merge method is an *insertion*, where the unaligned node has descendants that are in S. This always occurs when S' has greater resolution than S. For example, see Fig. 2, where WoRMS provides greater resolution than NCBI.

The vast majority of alignment and merge situations are simple, similar to the examples shown in Fig. 2. However, even a small fraction of special cases can add up to thousands when the total number of alignments and merges measures in the millions, so we have worked to develop heuristics that handle the most common special cases. Ambiguities caused by homonym name-strings create most of the difficulties, with inconsistent or unclear higher taxon membership creating the rest. The development of the assembly process described here has been a driven by trial and error - finding cases that fail and then adding or modifying alignment heuristics and other logic to address the underlying cause. Because the sources are noisy and inconsistent, any automated assembly process will make mistakes. To prevent or correct these mistakes, manual *ad hoc* adjustments are applied as needed, as a last resort. The goal in method development is to keep the number of needed adjustments small.

### Taxonomic sources

We build the taxonomy from ten sources. Some of these sources are from taxonomy projects, while others were manually assembled based on recent publications. As described above, OTT assembly is dependent on the input order of the sources - higher ranked inputs take priority over lower ranked inputs. Table 1 lists the sources used to construct OTT. The full provenance details, and a copy of the normalized source, are available in supplementary data.

**Open Tree curation** : It is not uncommon to have taxa as OTUs in phylogenetic studies that do not occur in OTT. This can be due to a delay in curation by the source taxonomy, a delay in importing a fresh source version into OTT, a morphological study containing otherwise unknown species, or other causes. To handle this situation, we developed a user interface that allows curators to create new taxon records along with relevant documentation (publications, databases, and so on). New taxon records are saved into a public GitHub repository, and these records are then linked from the OTT taxonomy files and user interfaces so that provenance is always available.

**Separation taxa** : This is a small curated tree containing 31 major groups such as Fungi, Metazoa, and Lepidoptera. Its purpose is to assist in alignment, where homonym name-strings are, or might need to be, present. If a node is found in one of these separation groups, then it will not match a node in a disjoint separation group, absent other evidence (details below).

**ARB-SILVA taxonomy processing** : The terminal taxa in the SILVA taxonomy are algorithmically generated clusters of RNA sequences derived from GenBank records. Rather than incorporate these idiosyncratic, fine-grained groupings into OTT, we use sequence record metadata to place the clusters into larger groups corresponding to NCBI taxa, and include those larger groups in OTT.

We excluded SILVA's plant, animal, and fungal branches from OTT because these groups are well covered by other sources and poorly represented in SILVA. For example, SILVA has only 299 taxa in Metazoa, compared with over 500,000 taxa under Metazoa in NCBI Taxonomy.

**Extinct / extant annotations** : Curators requested information about whether taxa were extinct vs. extant. With the exception of limited data from WoRMS and Index Fungorum, this information was not explicitly present in our other sources, so we imported IRMNG, which logs the extinct / extant status of taxa.

As a secondary heuristic, records from GBIF that originate from PaleoDB, and do not come from any other taxonomic source, are annotated extinct. This is not completely reliable, as some PaleoDB taxa are extant.

**Suppressed records** : We suppress the following source taxonomy records:

- animals, plants, fungi in SILVA
- GBIF backbone records that originate from IRMNG (IRMNG is imported separately)
- GBIF backbone records that originate from IPNI
- GBIF backbone records whose taxonomic status is 'doubtful'
- GBIF backbone records for infraspecific taxa (subspecies, variety, form)
- IRMNG records whose nomenclatural status is 'nudum', 'invalid', or any of about 25 similar designations
- NCBI Taxonomy records that have no potential for unification with OTUs in phylogenetic studies: those with name-strings containing 'insertion sequences', 'artificial librarries', 'transposons', or any of about 15 similar designations

The IPNI and IRMNG derived GBIF records are suppressed because they include many invalid names. We pick up most of the valid names from other sources, such as the direct IRMNG import, so this is not a great loss. Although GBIF's original taxonomic sources indicate which names are known to be invalid, this information is not provided by the GBIF backbone. Note that the GBIF backbone might import the same name from more than one source, but its provenance information only lists one of the sources. We suppress the record if that one source is IPNI or IRMNG.

**Sources not included** : The number of sources was of course limited by the amount of time we had available for import efforts; new sources were only added for specific reasons related to curators' interests. The choice of sources was also limited by the open data goal. Certain obvious choices, such as Catalog of Life, had to be passed over because there was no access, or access was controlled by legal terms of use.

### Import and Normalization

Each source taxonomy has its own import procedure, usually a file download from the provider's web site followed by application of a script that converts the source to a common internal form for import (a set of nodes, see terminology section). Given the converted source files, the taxonomy can be read by the OTT assembly procedure.

After each source taxonomy is loaded, the following normalizations are performed:

1. Diacritics removal - accents, umlauts, and other diacritic marks are removed in order to improve name matching, as well as to follow the nomenclatural codes, which prohibit them. The original name-string is kept.
2. Child taxa of "containers" in the source taxonomy are made to be children of the container's parent. "Containers" are groupings in the source that don't represent taxa, for example nodes named "incertae sedis" or "environmental samples". The members of a container aren't more closely related to one another than they are to the container's siblings; the container is only present as a way to say something about the members. The fact that a node had originally been in a container is recorded as a flag on the child node.
3. When a subgenus X has the same name-string as its containing genus, its name-string is changed to "X subgenus X". This follows a convention used by NCBI Taxonomy and helps distinguish the two taxa later in assembly.
4. Sibling taxa with the same name-string are combined.

The normalized versions of the taxonomies then become the input to subsequent processing phases.

### Aligning nodes across taxonomies

This section and the next give details of the taxonomy combination method introduced above.

OTT is assembled in a temporary work area or *workspace* by alternately aligning a source to the workspace and merging that source into the workspace. It is important that source taxonomy nodes be matched with workspace nodes when and only when this is appropriate. A mistaken identity between a source node and a workspace node can be disastrous, leading not just to an incorrect classification but to downstream curation errors in OTU matching (e.g. putting a snail in flatworms). A mistaken non-identity (separation) can also be a problem, since taxon duplication (i.e. multiple nodes for the same taxon) leads to loss of unification opportunities in tree synthesis.

As described above, source taxonomies are processed (aligned and merged) in priority order. For each source taxonomy, *ad hoc* adjustments are applied before automatic alignments. For automatic alignment, alignments closest to the tips of the source taxonomy are found in a first pass, and all others in a second pass. The two-pass structure permits first-pass alignments to be used during the second pass (see Overlap, below).

### Merging unaligned source nodes

After the alignment phase, we are left with the set of source nodes that could not be aligned to the workspace. The next step is to determine if and how these (potentially new) nodes can be merged into the workspace.

The combined taxonomy (U, above) is constructed by adding copies of unaligned nodes from the source taxonomy S' one at a time to the workspace, which initially contains a copy of S. Nodes of S' therefore correspond to workspace nodes in either of two ways: by mapping to a copy of an S-node (via the S'-S alignment), or by mapping to a copy of an S'-node (when there is no S'-S alignment for the S'-node).

As described above, each copied S'-node is part of either a graft or an insertion. A graft or insertion rooted at r' is attached to the workspace as a child of the nearest common ancestor node of r''s siblings' images. A graft is flagged *incertae sedis* if that NCA is a node other than the parent of the sibling images. By construction, insertions never have this property, so an insertion is never flagged *incertae sedis*.

**Figure 3.**
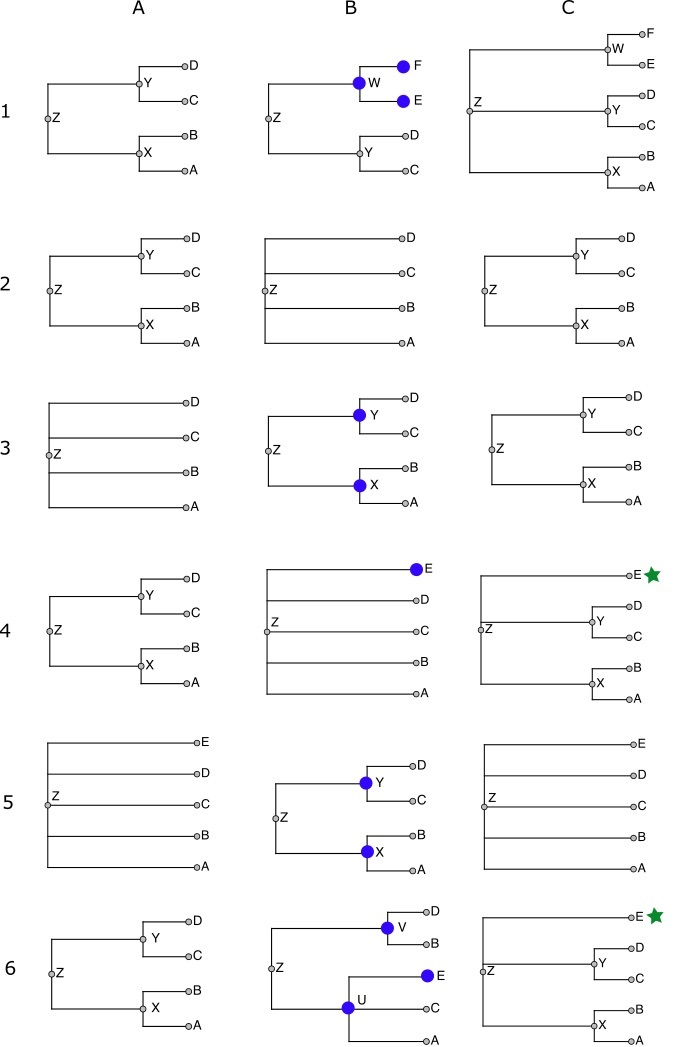
Merging taxonomies: *Examples* of outcomes when merging nodes from a source taxonomy into the workspace taxonomy. Each row (1-6) corresponds to one of the six examples described in the text. Column A is the current workspace taxonomy, column B is the source taxonomy being merged, and column C is the resulting workspace taxonomy. Nodes in B marked with a large blue circle are those that cannot be aligned to the workspace. Nodes in C marked with a green star are those flagged as *incertae sedis* in the final taxonomy.

The following schematic examples illustrate each of the cases that come up while merging taxonomies. Taxonomy fragments are written in Newick notation (Olsen 1990). Fig. 3 illustrates each of these six cases.

Case 1: ((a,b)x,(c,d)y)z + ((c,d)y,(e,f)w)z = ((a,b)x,(c,d)y,(e,f)w)z

This is a simple graft. The taxon w does not occur in the workspace, so it and its children are copied. The workspace copy of w is attached as a sibling of its siblings' images: its sibling is y in S', which is aligned to y in the workspace, so the copy becomes a child of y's parent, or z.

Case 2: ((a,b)x,(c,d)y)z + (a,b,c,d)z = ((a,b)x,(c,d)y)z

No nodes are copied from S' to the workspace because every node in S' is aligned to some node in S - there are no nodes that *could* be copied.

Case 3:(a,b,c,d)z + ((a,b)x,(c,d)y)z = ((a,b)x,(c,d)y)z

Supposing x and y are unaligned, then x and y from S' insert into the classification of z. The workspace gets copies of these two S'-nodes.

*Example*: superfamily Chitonoidea, which is in WoRMS (S') but not in NCBI Taxonomy (S), inserts into NCBI Taxonomy. Its parent is suborder Chitonina, which is in NCBI (i.e. aligned to the workspace), and its children are six families that are all in NCBI (aligned).

Case 4: ((a,b)x,(c,d)y)z + (a,b,c,d,e)z = ((a,b)x,(c,d)y,?e)z

In this situation, we don't know where to put the unaligned taxon e from S': in x, in y, or in z (sibling to x and y). The solution used here is to add e to z and mark it as *incertae sedis* (indicated above by the question mark).

For example, family Melyridae from GBIF has five genera, of which two (*Trichoceble*, *Danacaea*) are not found in the workspace, and the other three do not all have the same parent after alignment - they are in three different subfamilies. *Trichoceble* and *Danacaea* are made to be *incertae sedis* children of Melyridae, because there is no telling which NCBI subfamily they are supposed to go in.

Case 5: (a,b,c,d,e)z + ((a,b)x,(c,d)y)z = (a,b,c,d,e)z

We don't want to lose the fact from the higher priority taxonomy S that e is a proper child of z (i.e. not *incertae sedis*), so we discard nodes x and y, ignoring what would otherwise have been an insertion.

So that we have a term for this situation, say that x is *absorbed* into z.

Case 6: ((a,b)x,(c,d)y)z + ((a,c)p,(b,d,e)q)z = ((a,b)x,(c,d)y,?e)z

If the source has a hierarchy that is incompatible with the one in the workspace, the conflicting source nodes are ignored, and any unaligned nodes (e) become *incertae sedis* nodes under an ancestor containing the incompatible node's children.

For example, when WoRMS is merged, the workspace has, from NCBI,

((Archaeognatha)Monocondylia,(Pterygota,Zygentoma)Dicondylia)Insecta

and the classification given by WoRMS is

((Archaeognatha,Thysanura=Zygentoma)Apteryogota,Pterygota)Insecta

That is, NCBI groups Thysanura (Zygentoma) with Pterygota, while WoRMS groups it with Archaeognatha. The WoRMS hierarchy is ignored in favor of the higher priority NCBI hierarchy. If Insecta in WoRMS had had an unaligned third child, it would have ended up *incertae sedis* in Insecta.

The test for compatibility is very simple: a source node is incompatible with the workspace if the nodes that its aligned children align with do not all have the same parent.

### Final patches

After all source taxonomies are aligned and merged, we apply general *ad hoc* additions and patches to the workspace, in a manner similar to that employed with the source taxonomies. Patches are represented in three formats. An early patch system used hand-written tabular files, additions via the user interface use a machine-processed JSON format, and most other patches are written as simple Python statements. There are 106 additions in JSON form, 97 additions and patches in tabular form, and approximately 121 in Python form.

### Assigning identifiers

The final step is to assign unique, stable identifiers to nodes so that external links to OTT nodes will continue to function correctly after the previous OTT version is replaced by the new one.

Identifier assignment is done by aligning the previous version of OTT to the new version. As with the other alignments, there are scripted *ad hoc* adjustments to correct for some errors that would otherwise be made by automated assignment. For this alignment, the set of heuristics is extended by adding rules that prefer candidates that have the same source taxonomy node id as the previous version node being aligned. After transferring identifiers of aligned nodes, any remaining workspace nodes are given newly 'minted' identifiers.

The alignment is computed only for the purpose of assigning identifiers; the previous OTT version is not merged into the workspace. An identifier can only persist from one OTT version to the next if it continues to occur in some source taxonomy.

## Results

The assembly method described above yields the reference taxonomy that is used by the Open Tree of Life project. The taxonomy itself, the details of how the assembly method unrolls to generate the taxonomy, and the degree to which the taxonomy meets the goals set out for it are all of interest in assessing how, and how well, the method works. We will address each of these three aspects of the method in turn.

### Summary of Open Tree Taxonomy

The methods and results presented here are for version 3.0 of the Open Tree Taxonomy (which follows five previous releases using the automated assembly method). The taxonomy contains 3,594,550 total taxa; 3,272,177 tips; and 277,365 internal nodes. 2,335,412 of the nodes have a Linnean binomial of the form *Genus epithet*. There are 1,842,403 synonym records and 9,089 name-strings that are primary for more than one nodes. A longer list of metrics is in Table 2.

The number of species level homonym name-strings (2867) is surprisingly high. While a small number of these are legitimate, e.g. *Scoparia dulci* which is used in practice for both a plant and an insect, most of them result from errors in sources. Some originate in a single source, but most seem to be between nodes contributed by multiple sources. We researched ten cases chosen at random, and in every one, two sources disagreed on placement in separation taxa, and only one source is correct. E.g. *Callirhynchius exquisitus* is in beetles in one source and in decapods in another, so the Separation heuristic prevents alignment, and the workspace ends up with two nodes. But the decapod placement is incorrect, and there is really only one species. (Amazingly, every one of the ten samples was later corrected in the source database!)

### Results of assembly procedure

**Figure 4.**
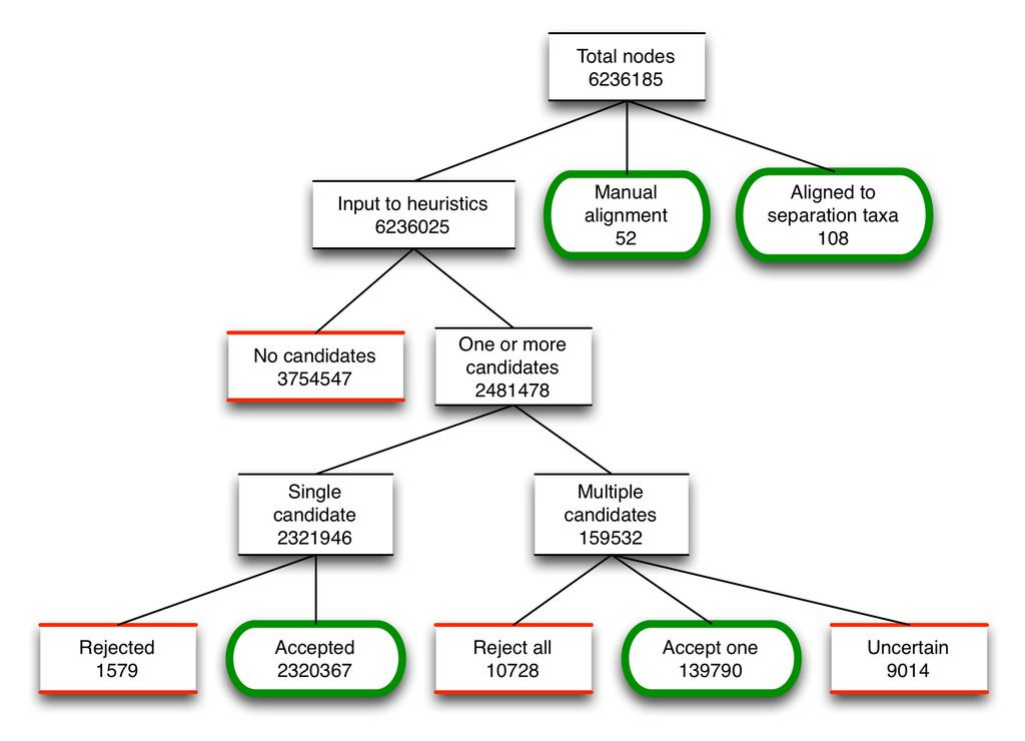
Fate of nodes as they move through the alignment procedure. Green, rounded boxes are endpoints that result in aligned nodes, while red, sqaure boxes are endpoints that result in unaligned nodes.

As OTT is assembled, the alignment procedure processes every source node, either choosing an alignment target for it in the workspace based on the results of the heuristics, or leaving it unaligned. Fig. 4 illustrates the action of the alignment phase. The presence of a single candidate node does not automatically align the two nodes - we still apply the heuristics to ensure a match (and occasionally reject the single candidate).

We counted the frequency of success for each heuristic, i.e. the number of times that a particular heuristic was the one that accepted the winning candidate from among two or more candidates. Table 3 shows these results. Separation (do not align taxa in disjoint separation taxa; used first), Lineage (align taxa with shared lineage; used midway through) and Same-name-string (prefer candidates who primary name-string matches; used last) were by far the most frequent.

After assembly, the next step in the method is to merge the unaligned nodes into the workspace taxonomy. Of the 3,780,949 unaligned nodes, the vast majority (99%) are grafted into the workspace. The remaining nodes (<1%) are either insertions, absorptions or remain unmerged due to ambiguities.

We also examined the fate of nodes from each of the input taxonomies, and Table 4 provides these results. The results are dependent on the order in which sources are added to the workspace. Overall, the number of conflicts is relatively low (<1%).

### Evaluating the taxonomy relative to requirements

The introduction sets out requirements for an Open Tree taxonomy. How well are these requirements met?

## Discussion

The primary actionable information in the source taxonomies consists of name-strings, and therefore the core of our method is a set of heuristics that can handle the common problems encountered when trying to merge hierarchies of name-strings. These problems include expected taxonomic issues such as synonyms, homonyms, and differences in placement and membership between sources. They also include errors such as duplications, spelling mistakes, and misplaced taxa. The problem cases add up to over 100,000 difficult alignments when the total number of source records measures over 6 million.

Ultimately there is no fully automated and foolproof test to determine whether two nodes can be aligned - whether node A and node B, from different source taxonomies, are about the same taxon. The information to do this is in the literature and in databases on the Internet, but often it is (understandably) missing from the source taxonomies.

It is not feasible to investigate such problems individually, so the taxonomy assembly methods identify and handle thousands of 'special cases' in an automated way. We currently use only name-strings, rudimentary classification information, and (minimally) ranks to guide assembly. We note the large role that our hand-curated "separation taxonomy" played in the alignment phase. This is a set of taxa that are consistent across the various sources, and allow us to make the (seemingly obvious) determination "these two taxa are in completely separate groups, so do not align them".

### Community curation

We have also developed a system for curators to directly add new taxon records to the taxonomy from published phylogenies, which often contain newly described species that are not yet present in any source taxonomy. These taxon records include provenance information, including references explaining the taxon, and the identity of the curator. We expose this provenance information through the web site and the taxonomy API.

We also provide a feedback mechanism on the synthetic tree browser, and find that most of the comments left are about presence, absence, choice, and spelling of labels, rather than the topology of the synthetic tree. These are issues that are addressed by improvements to the taxonomy. Expanding this feature to capture this feedback in a more structured, and therefore machine-readable, format would allow users to directly contribute taxonomic patches to the system.

### Comparison to other taxonomies

Given the unique goals of the Open Tree Taxonomy in comparison to most other taxonomy projects, it is difficult to compare OTT to other taxonomies in a meaningful way. The Open Tree Taxonomy is technically most similar to the GBIF taxonomy, in the sense that each is a synthesis of existing, overlapping taxonomies rather than a curated taxonomic database or one based on grafting. The GBIF method is yet unpublished (for basic information on the GBIF backbone see Döring 2016a, Döring 2016b). Once the GBIF method has been formally described, it will be useful to compare the two approaches and identify common and unique techniques for automated, scalable name-string matching and hierarchy merging.

### Potential improvements and future work

The development of the assembly software has been driven by the needs of the Open Tree project, not by any concerted effort to create a widely applicable or theoretically principled tool. A system like this is never finished, and this one is in its infancy. There are endless opportunities for bringing additional techniques, methods, data, and code libraries to bear, and we have faced difficult choices in deciding where to put our effort. Following are some of the directions for development that could have the highest impact.

- It is likely that alignment and merge could be improved by making better use of species proximity implied by the shape of the classification, and decreasing its reliance on the names of internal nodes. Better use of proximity might permit separation and identification of tips without use of a separation taxonomy, removing the need for the manual work of maintaining the separation taxonomy and the adjustment directives needed to align source taxonomies to it. An example is *Conolophus*, where two genus-level nodes in the same separation taxon are mistakenly combined. How to accomplish this is not obvious, but it is not obviously impossible.
- The alignment method should be extended to make use of authority information, when it is available. If name-strings match, or even if just species epithets match, then matching authority information is good evidence that the same taxon is meant. The form of authority information varies between sources, but could be normalized using the Global Names Parser (Patterson et al. 2016).
- Name-strings could also be analyzed to detect partial matches, e.g. matching on species epithets even when the genus disagrees, and spelling and gender variant recognition. Doing so would eliminate thousands of duplications. Other work on name matching, such as the Global Names Resolver (Patterson et al. 2016), goes far beyond what is done for OTT and these techniques should be used.
- The redescription problem described above should be addressed to the extent possible.
- Our handling of duplicate records in source taxonomies is incomplete and needs to be fixed. If two source nodes can be aligned to the same workspace node, then the duplication will not affect the workspace. But if there is no workspace node to align them to, the duplication persists in the workspace after the source taxonomy has been merged. There is special case logic when taxonomies are imported to fold together *sibling* duplicates, but not alignable duplicates generally.
- An assembly run can lead to a variety of error conditions and test failures. Currently these are difficult to diagnose, mainly for lack of technology for displaying the particular pieces of the sources, workspace, and assembly history that are relevant to the error. Once this information is surfaced it is usually not too difficult to work out a fix in the form of a patch or an improvement to the program logic. A small amount of automation could speed this kind of investigation and save curator time.
- The community curation should be developed, as mentioned above. Its success would depend on allowing users to test proposed changes and diagnose and repair any problems with them.
- Curators frequently request new taxonomy sources. The most frequently requested are improved fish, bird, plant, and paleontological sources. Community members have also suggested the Plazi TreatmentBank (Miller et al. 2015). Again, the information is generally available, but not yet harvested. (Some frequently requested sources may only be accessed under agreement with contractual terms (variously called "terms of use" or a "data use agreement"). One of these is the IUCN Red List (International Union for Conservation of Nature and Natural Resources 2016), an important source of up-to-date information on mammal species. These sources are off limits to Open Tree due to the project's open data requirement.)
- The presence of invalid and unaccepted names remains a significant problem. The information needed to detect them is available, and could be harvested.
- Basic usability features for application to new projects would include proper packaging of the application, and support for Darwin Core (Wieczorek et al. 2012) for both input and output.

Future work on taxonomy aggregation should attempt a more rigorous and pluralistic approach to classification (Franz et al. 2016a, Kennedy et al. 2006, Lepage et al. 2014, Franz et al. 2016b). Alignment should detect and record lumping and splitting events, and the classification conflicts detected during merge should be exposed to users. Exposing conflicts is in the interest of scientific transparency. Retaining alternative groupings could be useful in phylogenetic analysis, as a check on which of the sources agree or disagree with a given analysis. Lumping and splitting due to redescription, which lead to the same name-string (including author) referring to different taxa in different sources, could be recorded using multiple nodes qualified by description or source ('*sensu*'). Ideally, better handling of descriptions in aggregators ought to encourage sources to make links to primary sources more readily available for a variety of purposes.

## Data resources

All source code is open source (licensed BSD 2-clause) and available on GitHub at https://github.com/OpenTreeOfLife/reference-taxonomy. A snapshot of the code used to produce the version of OTT described here is archived at Zenodo (https://doi.org/10.5281/zenodo.546111). All data, including Open Tree Taxonomy 3.0 and all processed source taxonomies is archived on Dryad (cannot be uploaded before paper accepted; version for review at https://github.com/OpenTreeOfLife/reference-taxonomy/tree/master/doc/method/data-package).

## Conclusions

We have presented a method for merging multiple taxonomies into a single synthetic taxonomy. The method is designed to produce a taxonomy optimized for the Open Tree of Life phylogenetic tree synthesis project. Most taxonomy projects are databases of taxonomy information that are continuously updated by curators as new information is published in the taxonomic literature. In contrast, the Open Tree Taxonomy takes several of these curated taxonomies and assembles a synthetic taxonomy *de novo* each time a new version of the taxonomy is needed.

We have also developed a system for curators to directly add new taxa to the taxonomy from published phylogenies. These taxon additions include provenance information, including the source of the taxon and identity of the curator. We expose this provenance information through the website and the taxonomy API. Most of the Open Tree feedback has been about taxonomy, and expanding this feature to other types of taxonomic information allows users to directly contribute expertise and allows projects to easily share that information.

Taxonomic information is certainly best curated at a scale smaller than "all life" by experts in a particular group. Therefore, producing comprehensive taxonomies is always a synthesis of curated taxonomies. We advocate for the type of methods being used by Open Tree and by GBIF, where synthesis of overlapping sources is done in a repeatable fashion from sources, allowing changed information in sources to be quickly included in the comprehensive taxonomy, and also allowing continuous improvement to the synthesis method. Provenance information is retained and presented as part of the synthetic taxonomy. This type of synthesis requires that source taxonomies be available online, either through APIs or by bulk download, in a format that can be easily parsed, and ideally without terms of use that prevent distribution and reuse of the resulting synthetic taxonomies.

## Figures and Tables

**Table 1.** List of taxonomic sources: The ten sources used in v3.0 of Open Tree Taxonomy. Six are online taxonomic resources (SILVA (Quast et al. 2013), Index Fungorum (Index Fungorum Partnership 2014), WoRMS (WoRMS Editorial Board 2015), NCBI, GBIF, IRMNG), two are from publications (Hibbett et al. 2007, Schäferhoff et al. 2010), one is a small curated taxonomy of major groups to aid in assembly ("separation taxa"), and one consisting of taxonomic additions from phylogenies input into the Open Tree system ("Open Tree curation"). See text for explanation of 'Open Tree curation' and 'separation taxa'. Detailed provenance information for each source can be found in the accompanying data package. 'Focus' refers to the taxa of interest to Open Tree curators motivating inclusion in assembly. Key to 'reasons' column: O = added in order to improve OTU coverage; P = added in order to improve phylogenetic classification; T = added in order to improve taxonomic coverage.

Name	OTT Focus	Taxa	Synonyms	Priority order	Reasons
separation taxa	life	31	8	1	
SILVA	life	78687	0	2	P
Hibbett 2007	Fungi	227	0	3	P
Index Fungorum	Fungi	284973	157734	4	P,T
Schäferhoff 2010	Lamiales	119	0	5	P
WoRMS	Malacostraca, Cnidaria	330412	223196	6	P,T
NCBI	life	1488029	719526	7	O,P,T
GBIF	life	3273321	1143026	8	T
IRMNG	life	1706655	685983	9	T
Open Tree curation	n/a	n/a	n/a	10	O

**Table 2.** Summary of Open Tree Taxonomy 3.

**Number nodes**	**Property**
3594550	Total taxon records (nodes)
1842403	Synonym records
277365	Internal (non-tip) nodes
3272177	Tips.
3116485	Rank of 'species'
70886	Below the rank of species (e.g. subspecies, variety)
67070	Above the rank of species that subtend no node of rank species
2335412	Name-string has the form of a Linnaean binomial Genus epithet
9089	Homonym name-strings
2867	Homonym name-strings where the nodes have species rank
6110	Homonym name-strings where the nodes have genus rank
38	Maximum nesting depth of any node in the taxonomy
53287	Maximum number of children for any node in the taxonomy
12.96	Branching factor (average number of children per internal node)
45008	Source taxa that were absorbed into a larger taxon
317624	Marked *incertae sedis* or equivalent
252600	Annotated as being for an extinct taxon

**Table 3.** Frequency of success of alignment heuristics. In cases where there were multiple candidate nodes, this table lists the number of times that a particular heuristic was the one to select a single candidate. Heuristics are listed in the order in which they are applied. Success of an ealier heuristics means that a later heuristic is not used for a given node.

Alignment heuristic	Number nodes
Separation	22126
Disparate ranks	154
Lineage	25688
Overlap	7438
Proximity	228
Same name-string	84211

**Table 4.** Fate of source nodes from each of the input taxonomies. Unaligned nodes are either copied into the workspace or absorbed. Aligned nodes are added to the workspace through grafting or insertion.

**Source**	**Total**	**Copied**	**Aligned**	**Absorbed**	**Conflict**
separation	30	30	0	0	0
SILVA	74400	74395	5	0	0
Hibbett 2007	227	226	1	0	0
Index Fungorum	276262	276048	188	25	1
WoRMS	327570	269029	57026	1283	232
Schäferhoff 2010	119	118	1	0	0
NCBI	1320665	1198221	119532	2441	471
GBIF	2451566	1640700	808757	1963	146
IRMG	1561123	90746	1466929	3128	320
curated	29	29	0	0	0
total	6011991	3549542	2452439	8840	1170

## Hosting institution

Duke University

## Hosting institution

Duke University
